# A quick and simple spectrophotometric method to determine total carbon concentrations in root exudate samples of grass species

**DOI:** 10.1007/s11104-022-05519-w

**Published:** 2022-06-04

**Authors:** Eva Oburger, Christiana Staudinger, Andreea Spiridon, Vera Benyr, David Aleksza, Walter Wenzel, Michael Santangeli

**Affiliations:** 1 Department of Forest and Soil Science, Institute of Soil Research, University of Natural Resources and Life Sciences, Tulln, Austria; 2 Department of Chemistry, Institute of Analytical chemistry, University of Natural Resources and Life Sciences, Vienna, Austria

**Keywords:** Rhizodeposition, Metabolomics, Root exudation, Dissolved organic carbon, Carbon dynamics, Rhizosphere

## Abstract

**Purpose:**

Root exudates are key components driving belowground interaction between plant, microbes and soil. High-end analytical approaches provide advanced insights into exudate metabolite diversity, however, the amount of total carbon (C) released by roots should always be determined as the most basic parameter when characterizing root exudation as it (i) provides quantitative information of C exuded into the surrounding soil and (ii) allows to relate the abundance of individual exudate compounds to total C released. Here we propose a simple and quick, spectrophotometry-based method to quantify total dissolved organic carbon (DOC) concentration in exudation samples that is based on measuring the absorption of a pre-filtered but otherwise untreated exudate sample at 260 nm (DOC_260_).

**Method:**

Exudate samples collected from different grass genotypes (*Zea mays, Oryza sativa, Hordeum vulgare)* grown in various experimental settings (soil, hydroponic) were analysed with the DOC_260_ assay and results were compared with C concentrations obtained by liquid TOC-analyser.

**Conclusion:**

We demonstrated that the DOC_260_ method allowed for quick and inexpensive measurements of total dissolved organic carbon concentrations in exudate samples from grass species grown under nutrient sufficient as well as under P deficient conditions. Interestingly, DOC_260_ failed to predict DOC concentrations in exudate samples from plants grown under Zn and Fe deficiency suggesting a strong shift in metabolite composition under micronutrient deficiency. Even though the applicability of the DOC_260_ method remains to be tested on exudate samples originating from dicots and plants exposed to other environmental stresses (e.g. pathogen attack, heavy metal stress, etc), it will help to increase our understanding of root exudation and related rhizosphere processes in the future.

## Introduction

Roots release a large diversity of soluble or volatile organic molecules (i.e., root exudates) as well cell debris and sloughed-off root cap cells as they forage for water and nutrients. The functional importance of root exudates in driving belowground interactions between plant roots, soil microbes and soil matrix has been increasingly acknowledged in the last decades (e.g. Badri and Vivanco 2009; Coskun et al. 2017; Dennis et al. 2010; Sasse et al. 2018; Wen et al. 2022). Despite our awareness about the central role of exudates in rhizosphere processes, studies investigating exudate quality and quantity under natural growth conditions are still relatively scarce. The difficult choice of a suitable but also applicable exudate sampling procedure combined with challenging analytical approaches makes it easier to discuss the importance of exudates in soil than to actually investigate them (Oburger and Jones 2018). Most studies investigating root exudation in the past focused on individual compounds or compound classes (e.g. organic acids, amino acids, etc.), ignoring a large fraction of the total carbon (C) released by roots. Thanks to recent developments not only in analytical instrumentation, but also in computer power, available data processing software as well as metabolite data bases, the number of non-targeted metabolomic exudation studies aiming to reveal the entire metabolite composition released by roots has significantly increased in the past five years. While a more detailed understanding of exudate compound diversity will allow us to increasingly decipher the cross-talk between plants and microbes, it is important not to overlook the overall quantitative amount of carbon (C) released by roots. When focusing on specific exudate compounds using targeted analysis only, we miss the quantitative relevance of our target analytes compared to all other compounds released. Also, non-targeted metabolomics usually only allows the identification of a fraction of all metabolites released as available data bases are still limited, and non-targeted metabolomic analysis only provides qualitative information, or semi-quantitative at best. Logically, we focus on the identified compounds when interpreting and discussing our results. It is therefore of crucial importance to relate compound specific results to total C exudation to consider the general trend of C release in our data interpretation. Total C release rates are known to differ between species when grown under the same conditions (Canarini et al. 2016) but also within one genotype when grown on different soils/substrates (Oburger et al. 2014; Sasse et al. 2020). Furthermore, total C exudation rates (per unit root biomass or root surface area) has been found to decrease with increasing plant development (Santangeli et al. in prep.) These findings highlight the importance to measure total C exudation in addition to individual metabolite composition. From an ecological perspective, information of changes in total C release rates under changing environmental conditions is highly valuable, even if compound specific information is missing or available for only a few metabolites.

Here we propose a simple and quick, photometry-based method to quantify total dissolved organic carbon (DOC) concentrations in exudate samples. Our method was applied to liquid exudate samples, hence targeting the soluble fraction of exudates released. Based on a previous study applying photometry to estimate DOC concentrations in soil solutions, stem flow, throughfall and surface waters at 254 nm (Brandstetter et al. 1996), we tested the applicability of this approach to exudate samples. Our DOC assay was performed using a plate reader suitable for 96 microwell plates and calibrated using an internal calibration with potassium phthalate including independent quality controls on each individual plate with certified reference material traceable to standard reference material from NIST. Wavelength scans allowed us to optimize our assay by selecting a wavelength (i.e. 260 nm) that produced reliable results for total dissolved carbon concentrations in root exudation samples. To verify the suitability and reliability of our proposed photometry-based approach, we compared photometric results with results from DOC analysis by a liquid C analyser.

## Materials and methods

### Method development

#### Exudate samples

In total, 112 exudation samples from different experiments including experimental blanks as well as 3 independent quality controls (1, 5 and 10 mg C L^−1^, TOC Standard Solution 132,253, Supelco, Germany) were used for method development. All exudation samples tested were collected using either a hydroponic-only or a soil-hydroponic hybrid approach. While plants were grown in an artificial nutrient solution (half-strength Hoagland (Hoagland and Arnon 1950)) in the first experimental approach, the latter combined soil growth with careful root washing followed by a short hydroponic sampling period (Oburger and Jones 2018). Experimental plant species and specific genotypes, growth matrix and number of samples per growth matrix as well as corresponding growth and sampling conditions are summarized in Table 1. Plants were grown for different time periods prior to exudation sampling, either in the greenhouse or in growth cabinets. A limited number of samples from plants exposed to either P deficient conditions, Zn deficiency, or Fe deficiency (Table 1) Exudation sampling periods ranged between 2 and 3 hours. All exudates were sampled in Milli-Q water (MQW) containing either 0.005 or 0.01 g L^−1^ Micropur (Katadyn) to inhibit microbial decomposition of exuded metabolites from the sampling solution during the sampling process (Oburger et al. 2014). After the exudation sampling period, roots were removed from the sampling solution, samples were filtered using 0.2 μm cellulose acetate filters (OE 66, Whatman, UK) and then stored at −20 °C to await analysis.

**Table 1:** Overview of genotypes, growth as well as sampling conditions from which exudate samples were collected

*Growth matrix*	*Species*	*Genotype*	*Plant age at exudation sampling* *Days after seeding*	*Sampling volume (L)*	*Sampling period (h)*	*Nr samples per growth matrix*
High P Humic Haplic Andosol, pH_H2O_ 5.11, fertilized N:P:K at 150:150:150 mg kg^−1^	*Oryza sativa*	Nerica 4, DJ 123, Mudgo, IR 64	38	0.1	3	20
Low P Humic Haplic Andosol, pH_H2O_ 5.11, fertilized N:P:K at 150:10:150 mg kg^−1^	*O. sativa*	Mudgo, IR 64	38	0.1	3	10
Hydroponic - half-strength Hoagland	*Zea mays*	WT ^3^, *rth3* ^3^	22	0.5	2	11
Hydroponic - half-strength Hoagland	*Hordeum vulgare*	B13 Balashere, Bere JIC 4843	31	0.12	3	9
Hydroponic - half-strength Hoagland (-Zn)	*H. vulgare*	Bere JIC 4843	31	0.12	3	5^d^
Hydroponic - half-strength Hoagland (-Fe)	*H. vulgare*	Bere JIC 4843	31	0.12	3	5^d^
Quartz sand loam mix^1^	*Z. mays*	WT ^3^, *rth3* ^3^	22	0.5	2	12
Loam^1^	*Z. mays*	WT ^3^, *rth3* ^3^	22	0.5	2	28
Experimental blanks^2^						12
Total sum of samples						**112**

^1^substrate characteristics published in Vetterlein et al. (2021)

^2^MWQ water plus Micropur (10 mg L^−1^) from different experiments

^3^see also Vetterlein et al. (2021); Wen and Schnable (1994)

^4^samples were excluded from regression

#### Analysis of total dissolved organic carbon using a liquid TOC analyser

A calibration for total dissolved organic carbon ranging from 0.5 to 30 mg C L^−1^ was prepared using potassium phthalate (KHP, Elementar 35.00–0151) dissolved in MQW water. Aliquots (5 mL) of standards, blanks and exudation samples were analysed for total dissolved organic C by a Vario Elementar TOC analyser (Elementar Analysensysteme GmbH, Germany) for liquid samples. Determination of C concentrations in samples was based on a high temperature catalytic oxidation approach, including the combustion of samples at 850 °C followed by the determination of CO_2_ by an infrared CO_2_ detector. Prior to analysis, 40 μL of 10% HCl were added to each sample to ensure degassing of inorganic carbonates from the solution. However, tests revealed that in contrast to soil solution samples, inorganic carbonates are negligible in exudation samples (data not shown). Obtained results were then used to optimize and verify the suitability of our DOC photometric assay.

#### Testing photometric absorption to determine total dissolved organic carbon concentrations in exudate samples

The same calibration standard solution used for the TOC analysis was also used in the photometric wavelength scan and both methods were carried out on the same day to avoid any effects of repeated freezing/thawing of the exudate samples. Calibration blanks, calibration standards as well as thawed exudation samples and sample blanks (250 μL) were pipetted into 96 microwell plate (Greiner UV-STAR® flat-bottom) suitable for absorbance analysis in the UV/VIS spectrum. Next, these plates were scanned using a Tecan Infinite 200 PRO (Tecan, Switzerland). For method development and optimization, a wavelength scan from 230 nm to 370 nm was applied to all samples, blanks, and calibration standards. Obtained results where then compared against results obtained by the liquid TOC analyser. Simple linear regression plots and related statistical parameters were determined using Graphpad Prism 9.3.1 for Windows (GraphPad Sofware, San Diego, CA, USA) and r^2^ was calculated to evaluate the goodness of fit of the linear regression model.

### Final photometric assay to determine total dissolved organic carbon concentrations in exudate samples

Prepare a calibration for total dissolved organic carbon ranging from 0 to 30 mg C L^−1^ using potassium phthalate (KHP, Elementar 35.00–0151) dissolved in MQW water as well as two independent quality control solutions (TOC Standard Solution 132,253, Supelco, Germany). Pipette calibration blanks, standards, quality controls as well as exudate samples and respective sample blanks (250 μL) into 96 well plate (e.g. Greiner UV-STAR® flat-bottom) suitable for absorbance analysis in the UV/VIS spectrum and measure absorbance at 260 nm.

## Results

### Identifying a suitable wavelength for photometric determination of total C concentrations in root exudate samples

**Fig. 1:**
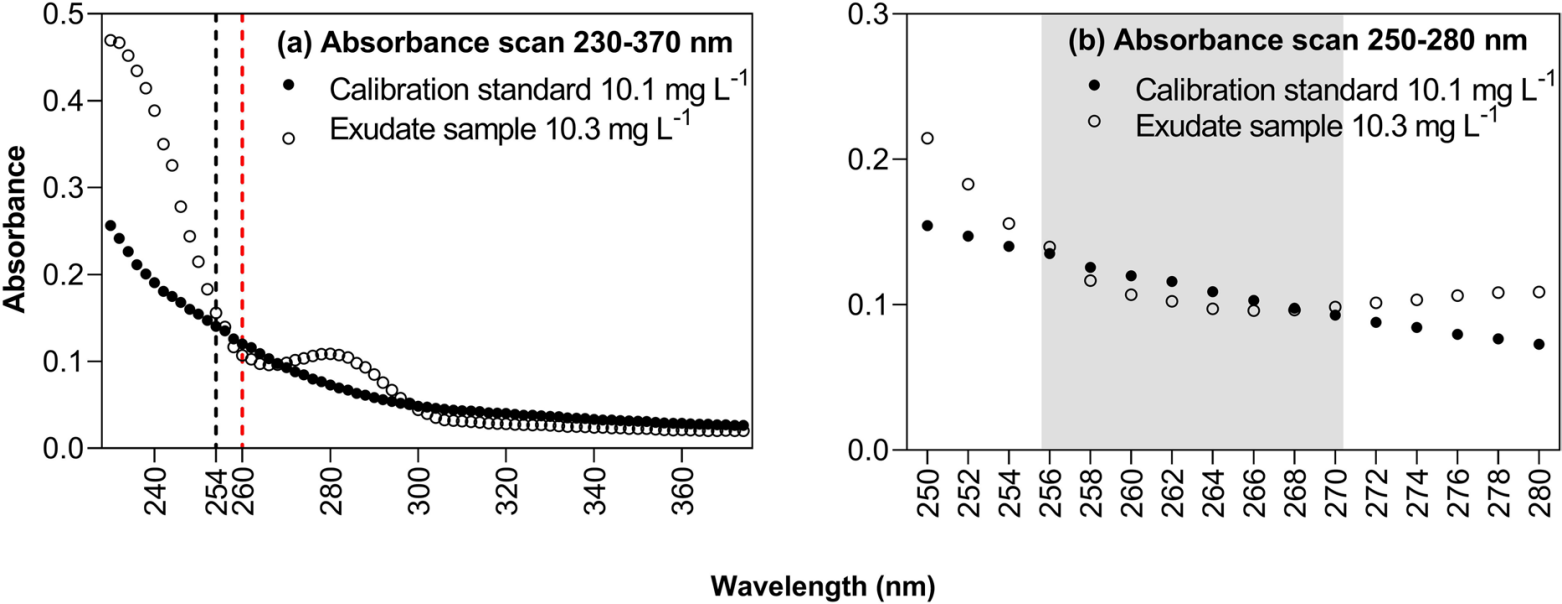
**a** Absorbance scans across the wavelength spectrum from 230 to 370 nm for one exemplary exudate sample (10.3 mg C L^−1^) and one calibration standard (10.1 mg C L^−1^). Red line indicates the selected wavelength (260 nm) for photometric DOC determination in exudation samples. **b** Absorbance scans across the wavelength spectrum from 250 to 280 nm. Grey area indicates most suitable wavelength spectrum (256–270 nm)

A comparison of wavelength scans of a calibration standard solutions (10.1 mg C L^−1^) and an exudation sample of similar concentration (10.3 mg C L^−1^) revealed differences in absorption behaviour across the investigated wavelength spectrum (230–370 nm, Fig. 1a) but showed a good fit at wavelengths 256–270 nm (Fig. 1b – highlighted in grey).

**Fig. 2:**
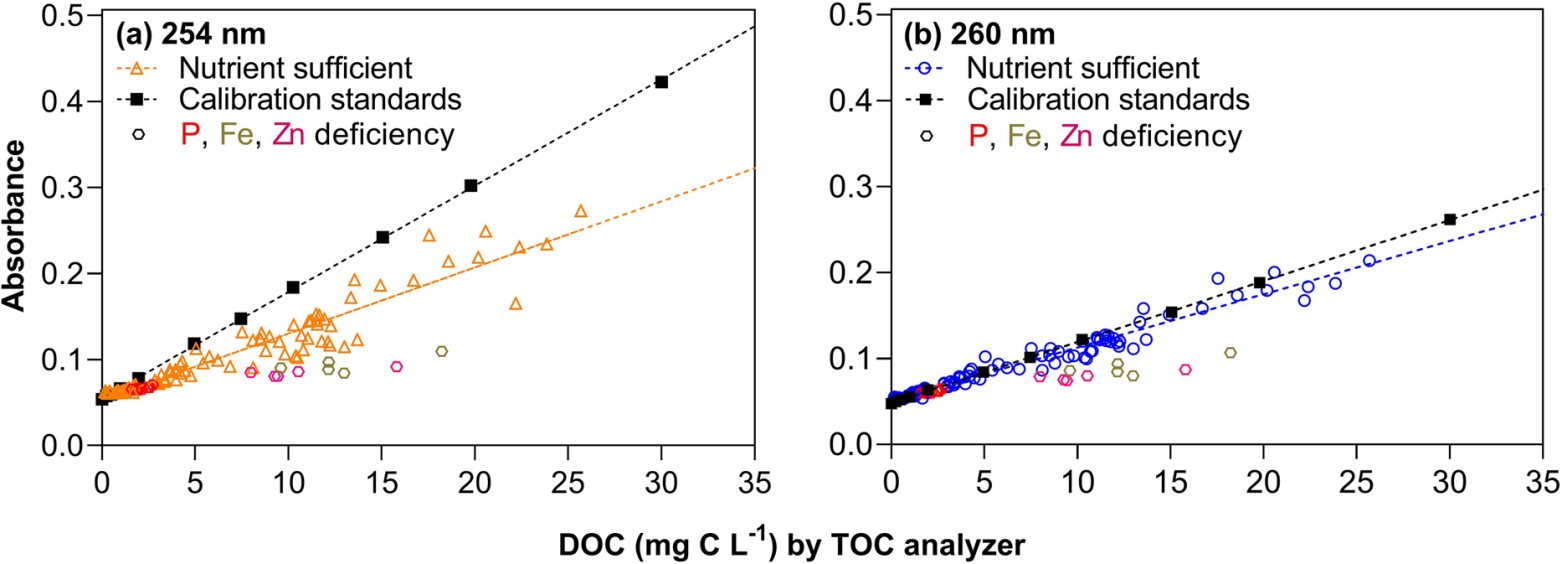
**a** Regression plot of absorbance measured in exudate samples and calibration standards at **254 nm** versus DOC concentrations determined separately by a liquid TOC analyser. **b** Regression plot of absorbance measured in exudate samples and calibration standards at **260 nm** versus DOC concentrations determined separately by a liquid TOC analyser

Regression plots of absorbance measured in exudate samples and calibration standards at 254 and 260 nm and the corresponding DOC concentrations determined separately by a liquid TOC analyser are presented in Fig. 2a, b. Both wavelengths resulted in a good correlation of absorbance values and TOC concentrations for plants grown under nutrient sufficient or P deficient conditions, however the slope differed significantly for DOC_254_ and DOC_260_ respectively. Interestingly, dissolved organic C concentration in exudate samples from grasses grown under Zn and Fe deficiency (Fig. 2a,b) were significantly underestimated irrespective of the wavelength applied. Consequently, those samples were excluded from the regression analysis.

Excluding samples from Fe and Zn deficient plants, we found that the slope of the standard curve measured at 260 nm was closest to the slope determined by plotting the wavelength specific absorption of exudate samples against the concentrations determined by the TOC analyser (Fig. 2b). In contrast and despite the higher sensitivity, the slope of calibration standards and exudate samples significantly differed at 254 nm (Fig. 2a). Regression analysis further confirmed that when measured at 254 nm, the slope of the calibration standards (0.0123) deviated from the slope obtained by the exudation samples (0.0077) (Table 2). In contrast, plotting the same data using the absorbance at 260 nm resulted in the most comparable slopes for exudate samples (0.0062) and calibration standards (0.0071) across all tested wavelengths (Fig. 2b, Table 2, results from other wavelengths tested are not shown). In addition, the correlation coefficient also revealed a more accurate prediction for DOC_260_ (r^2^ = 0.96) than for DOC_254_ (r^2^ = 0.91).

**Table 2:** Results from the linear regression models of absorbances of exudate samples (Abs Ex_260_ or Abs Ex_254_) plotted against the mg C L ^−1^ measured with the liquid TOC analyser and calibration standards plotted against the known standard concentration (Std_known_), with absorbance of exudate samples and standards measured at 254 (Abs Std_260_) and 260 nm. (Abs Std_260_)

	Abs Ex_260_ vs TOC	Abs Std_260_ vs Std_known_	Abs Ex_254_ vs TOC	Abs Sdt_254_ vs Std_known_
	Best-fit values			
Slope	0.0062	0.0071	0.0077	0.0123
Y-intercept	0.0508	0.0484	0.0533	0.055
1/slope	161.4	141.1	130.1	80.95
	95% Confidence Intervals			
Slope	0.0059 to 0.0064	0.0070 to 0.0071	0.0072 to 0.0082	0.0122 to 0.0125
Y-intercept	0.0485 to 0.0531	0.0477 to 0.0491	0.0489 to 0.0576	0.0531 to 0.0569
	Goodness of Fit			
*r*^*2*^	0.9586	0.9999	0.9090	0.9997
Equation	Y = 0.0062*X + 0.0508	Y = 0.0071*X + 0.0484	Y = 0.0077*X + 0.0533	Y = 0.0123*X + 0.055
*n*	102*	11	102*	11

*Fe and Zn deficient exudate samples were excluded from the regression model

**Fig. 3:**
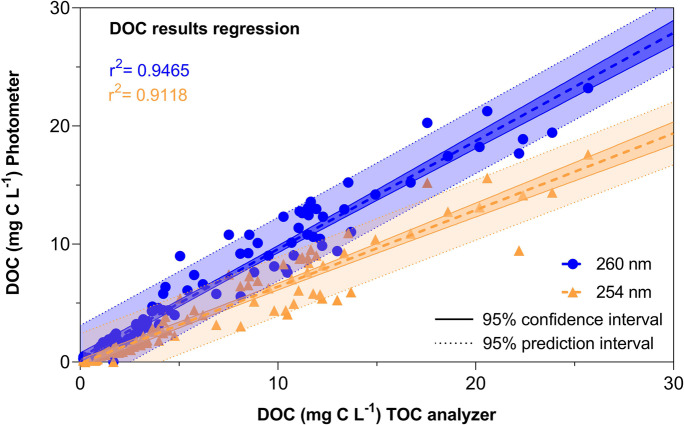
Regression plot of DOC concentrations of exudate samples (n = 102, excluding samples from Zn and Fe deficient plants) calibrated based on wavelength specific absorbance (254 nm, 260 nm) and determined by a liquid TOC analyser. Coloured areas within dotted lines represent the 95% prediction interval, while area within the continuous lines represent the 95% confidence interval

Applying the respective calibration equations obtained for both wavelengths (254 nm & 260 nm) to calculate DOC concentrations in our exudate samples and then plotting them against the concentrations separately determined by a liquid TOC analyser (Fig. 3, Table 3) revealed that the relationship between results obtained from TOC and DOC_260_ was nearly 1:1 (slope 0.92) while DOC_254_ underestimated TOC concentrations by about 30% (slope 0.65). This further supports the greater suitability of using 260 nm instead of 254 nm to reliable predict dissolved organic carbon concentrations in exudate samples.

**Table 3:** Results from the linear regression model of DOC measured at 260 and 254 nm versus the mg C L ^−1^ measured with the liquid TOC analyser

	DOC_260_ vs DOC_TOC_	DOC_254_ vs DOC_TOC_
	Best-fit values	
Slope	0.9167	0.6493
Y-intercept	0.3925	−0.1072
1/slope	1.091	1.540
	95% Confidence Intervals	
Slope	0.8735 to 0.9599	0.6092 to 0.6893
Y-intercept	0.0023 to 0.7827	−0.4689 to 0.2545
	Goodness of Fit	
*r*^*2*^	0.9465	0.9118
Equation	Y = 0.9671*X + 0.2708	Y = 0.7133*X - 0.09960
*n*	102	102

*Fe and Zn deficient exudate samples were excluded from the regression model

## Discussion and outlook

Here we tested and validated a quick and simple method to determine DOC concentrations in exudate samples by means of using a plate photometer in combination with 96-microwell plates suitable for UV/VIS light spectrum. Testing exudates from different grasses (barley, maize, rice) grown in different soils as well as in nutrient solution culture revealed that measuring the absorption of pre-filtered (0.2 μm) but otherwise unprocessed exudate samples at 260 nm is an accurate and fast approach to quantify total C (DOC) concentrations in exudate solutions from grass species grown under nutrient sufficient condition as well as under P deficiency. As our test samples only included monocotyledonous species, we currently cannot recommend the use of DOC_260_ approach for dicotyledonous species without additional testing. Furthermore, including exudate samples from barley grown under Zn or Fe deficiency revealed that while the DOC_260_ method worked well for exudate samples from the respective, fully fertilized control plants, it significantly underestimated total C exudate concentrations from Fe and Zn deficient plants. These results suggest a strong shift in metabolite composition exuded particularly under micronutrient (Fe, Zn) deficiency. This is supported by Marastoni et al. (2020) who observed distinct changes in metabolome fingerprints from root cuttings of two grapevine lines grown in hydroponics under Fe deficiency and the respective fully fertilized control conditions.

A previous study proposed 254 nm as the most suitable wavelength to determine DOC concentrations in soil solutions, stem flow and surface runoffs (Brandstetter et al. 1996). In contrast to this study, Brandstetter et al. (1996) did not include separate, independent calibration and reference standards in their photometric analysis but simply correlated results obtained by a TOC analyser with the absorbance measured at 254 nm. Correlating results from a TOC analyser with photometric absorption at 254 nm and 260 nm in our study revealed equally good fits for both wavelength (r^2^ = 0.96 and 0.91 respectively, Table 2). However, including independent calibration standards (Fig. 2a, b) and plotting results calibrated with the obtained calibration curves measured at 254 and 260 nm versus results from the TOC analyser (Fig. 3), showed that using 254 nm to determine total C in exudate samples underestimates C concentrations. Including independent calibrations standards and quality controls together with samples on each plate subjected to analysis by the plate photometer ensures not only accurate calibration of DOC concentrations but also allows to account for instrument specific variation as well as daily variation of instrument performance. Our approach is therefore more robust compared to published regression equations that might be affected by instrument performance. Whether or not our proposed method is also suitable for DOC analysis in soil solutions and potentially also superior to absorption values measured at 254 nm as proposed by Brandstetter et al. (1996) remains to be tested as this was out of scope in this study.

Measuring the absorbance at 254 nm has also been frequently used to characterize surface and soil water samples (Hu et al. 2017; Laurent et al. 2020; Weishaar et al. 2003; Welikala et al. 2021) and it has also been applied to root exudation samples (LeFevre et al. 2013). This method, generally referred to as SUVA_254 nm_ (specific UV absorbance at 254 nm), however, provides qualitative information about the degree of aromaticity of dissolved organic carbon and not quantitative information about the total dissolved C concentration.

Compound specific as well as non-targeted analytical approaches to capture species- and treatment-specific root exudation often require high end analytical equipment and time-consuming method development (van Dam and Bouwmeester 2016). Even though they deliver highly relevant in-depth information on exudation patterns, obtained results miss the quantitative relation of individual compounds, compound classes or metabolite fingerprints to total carbon exuded if DOC concentrations in exudate samples are not determined. Our proposed photometric assay (DOC_260_) holds promise to become an easily applicable standard method in exudate analysis as it enables experimentalists to accurately determine DOC concentrations in exudate samples with minimum effort using standard laboratory equipment (UV-photometer). Another benefit is the small sample volume (250 μL) required for the DOC_260_ analysis, as sample volume can be a critical factor in exudate research, particularly when individual root segments are sampled (Phillips et al. 2008) and/or sample pre-concentration is required (e.g. Oburger et al. 2014). Nevertheless, further tests are needed including dicot species and other growth conditions including other environmental stresses than the ones tested in this study (P, Zn, Fe deficiency) to assess the full application range of the DOC_260_ approach in exudate research. From an ecological point of view, quantitative information on total C exuded per plant if total root biomass is presented (mg or μmol C plant^−1^ h^−1^), or per unit root biomass or root surface area (mg or μmol C g root dry weight^−1^ h^−1^/ mg or μmol C g root surface area^−1^ h^−1^) is highly relevant as roots and exudation-fuelled rhizosphere processes are known to play a central role in soil carbon and nutrient cycling. Furthermore, the choice of exudation sampling approach and accurate data presentation is critical for obtaining ecologically relevant results that allow comparison between different studies (Oburger and Jones 2018). Increasing the number of data sets reporting C exudation rates by different plant species under different environmental conditions will not only increase our understanding about C exudation dynamics but will also improve the accuracy of model predictions of C dynamics in soils and ecosystems.
